# Development and clinical application of a high‐performance medical static computed tomography system

**DOI:** 10.1002/acm2.70456

**Published:** 2026-01-15

**Authors:** Haining Ding, Yunxiang Li, Zhili Cui, Hongchun Xu

**Affiliations:** ^1^ Department of Algorithm Nanovision Medical Technology (Shanghai) Co., Ltd Shanghai China

**Keywords:** array‐type integrated x‐ray source, photon stream detector, static CT, ultra‐high resolution

## Abstract

**Purpose:**

Conventional helical computed tomography (CT) is limited by constraints related to centripetal acceleration, with current rotation speeds nearing the boundaries of engineering feasibility. This study addresses these challenges by proposing a novel CT system design

**Methods:**

This study introduces an innovative multi‐source static CT architecture that employs an array‐based, fully integrated x‐ray source paired with a photon stream detector. This system utilizes a complete circular configuration and implements a sequential exposure strategy for each source, thereby eliminating the need for mechanical rotation typical of traditional helical CT. Leveraging the compact integration of the x‐ray source and the fine pixel structure of the detector, we developed a compressed‐sensing iterative reconstruction algorithm based on the bilateral extended Feldkamp‐Davis‐Kress (bixFDK), referred to as “iVision” reconstruction. In addition, a software‐based scatter correction algorithm was implemented. These enhancements collectively improve system performance by significantly boosting spatial resolution.

**Results:**

The multi‐source static CT system met all key regulatory standards for performance indicators, including image noise, uniformity, measurement accuracy, spatial resolution, and density resolution. Notably, the system achieved a spatial resolution of up to 25 LP/cm@MTF = 10%, positioning it at the forefront of ultra‐high‐resolution CT imaging. In volunteer clinical scans, the system consistently delivered sharper images of anatomical regions such as the head, chest, abdomen, and musculoskeletal structures, outperforming conventional helical CT, particularly in detailed visualization of bones and joints, pulmonary tissues, and internal organs.

**Conclusions:**

Based on the above results and analyses, this multi‐source static CT system achieves diagnostic‐quality imaging, aligning with clinical practice standards. It excels in capturing fine anatomical detail and detecting critical pathological features. Its high‐resolution capability also supports precise diagnoses of hip fractures and facilitates detailed trabecular bone assessment, thus improving overall diagnostic accuracy.

## 1 INTRODUCTION

Computed tomography (CT), renowned for its user‐friendly operation, rapid scanning capability, and high image quality, has become an indispensable tool across various domains, including clinical diagnostics,^1^, ^2^, ^3^ industrial quality assurance,^4^, ^5^ and security screening.^6^ The technology has undergone decades of evolution, beginning with the first‐generation systems employing pencil‐beam, translate‐rotate scanning, and advancing through innovations such as helical scanning, multi‐detector arrays,^7^ and spectral imaging.^8^ Among these advancements, third‐generation helical CT remains the most extensively adopted due to its technological maturity and practical utility. Despite its widespread use, this modality faces several critical limitations.

Mechanically, the slip‐ring system that enables gantry rotation has reached its performance ceiling.^9^, ^10^ These mechanical boundaries restrict further increases in rotation speed, thereby impeding improvements in spatial resolution and extending scanning times, constraints that are particularly detrimental in urgent diagnostic settings. Additionally, the high‐speed centrifugal motion exacerbates wear and tear on moving components, accelerating system degradation, increasing maintenance frequency and cost, and introducing risks of structural instability and operational vibration, all of which pose safety concerns.^11^, ^12^

On the design front, traditional helical systems employ a disjointed configuration, separating the x‐ray tube and high‐voltage generator. This architecture demands numerous independent components and intricate connections, resulting in elevated manufacturing costs and complicating installation, especially in spatially constrained environments. Furthermore, the need for separate inspection and repair of the tube and generator inflates maintenance complexity and duration. High‐voltage safety concerns further necessitate sophisticated protection mechanisms, consuming additional resources and technical input.^13^, ^14^, ^15^, ^16^

To address these entrenched limitations. We propose a high‐performance static CT system configured with a dual‐ring layout comprising an array‐based integrated x‐ray source and a photon stream detector. This design utilizes a multi‐source and multi‐detector structure, requiring only a small angle shifting.

Structurally, the system offers a distinct advantage. By meticulously optimizing the geometry and alignment of the source‐detector array, the x‐ray propagation path is refined, yielding more accurate and complete projection data. This geometric optimization enables the system to bypass the rotational speed barrier of traditional gantries,^17^, ^18^ significantly enhancing spatial resolution for fine‐detail imaging and shortening scan durations to suit rapid diagnostic and industrial workflows. Advanced materials and precision manufacturing have also been employed, strengthening system durability and operational stability. This not only minimizes wear common in rotating mechanisms but also ensures gantry rigidity and reduces vibration, mitigating safety risks.

In contrast to the bulky and maintenance‐heavy structure of conventional CT systems, the integrated layout introduced here streamlines the system, dramatically reducing production costs, equipment footprint, and installation complexity, making it better suited for diverse application environments. On the computational side, the system incorporates innovative algorithms and mathematical models to process the collected data efficiently and reconstruct images with high fidelity.

Altogether, static CT system blend of structural innovation and algorithmic advancement makes it a promising candidate for next‐generation imaging, enhancing diagnostic precision in clinical settings, increasing throughput in industrial inspections, and pushing the boundaries of what CT technology can achieve.

## 2 METHODS

### 2.1 Structural design

**FIGURE 1:**
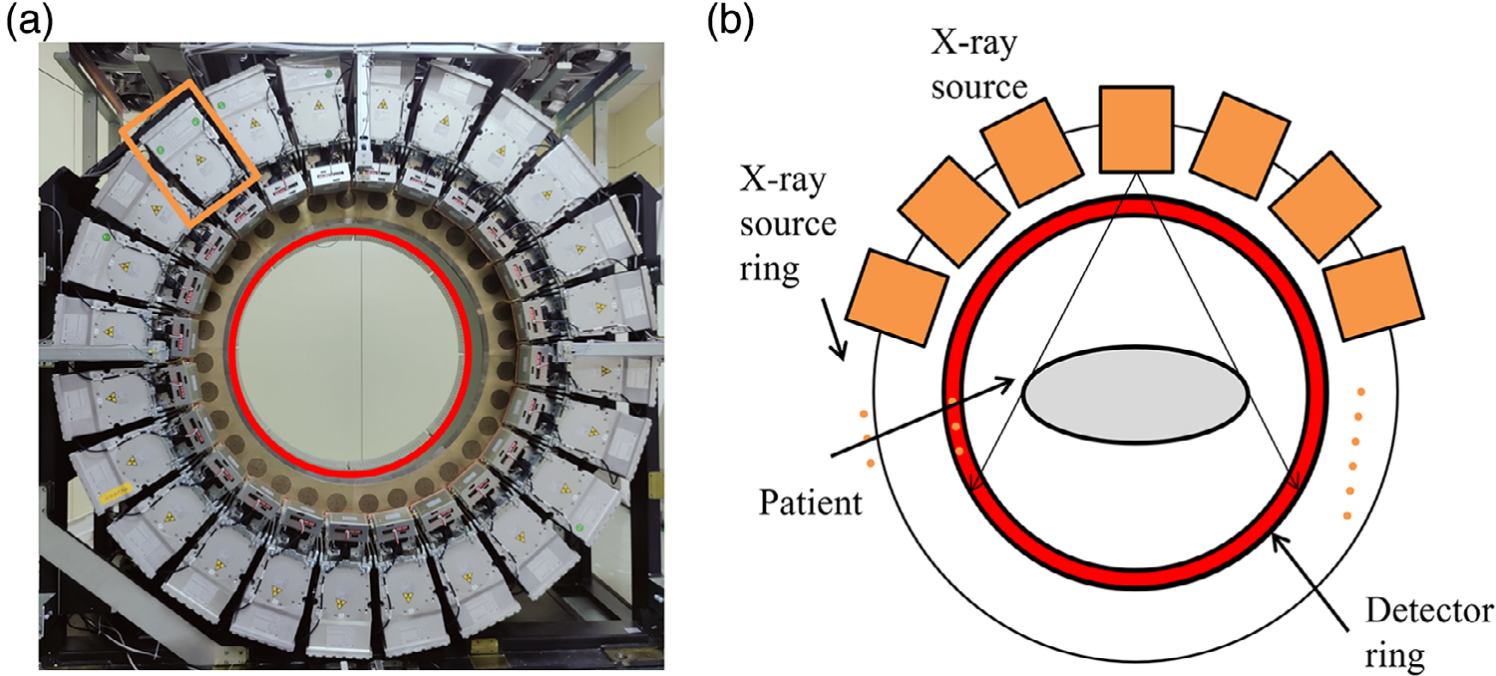
Photograph and schematic illustration of the static CT system. (a) Photograph of the static CT system; (b) Schematic diagram of the static CT system.

As shown in Figure 1, the static CT system features a dual‐ring architecture comprising a circular array of x‐ray sources and photon stream detectors. The outer ring houses 24 uniformly spaced integrated x‐ray sources, while the inner ring contains 64 evenly distributed photon stream detectors. This layout forms a novel dual‐ring configuration that is, firmly rooted in the core principles of CT imaging.

The underlying imaging mechanism adheres to the Lambert‐Beer attenuation law,^19^ mathematically expressed as $I_{\mathrm{out}}=I_{\mathrm{in}}\;e^{-\mu d}$, where μ is the x‐ray attenuation coefficient of the material, and *d* is the path length through the object. In clinical applications, as x‐rays pass through the human body, detectors capture the attenuated signals from various angles. These measurements are used to derive the attenuation coefficients of internal tissues, which are then converted into CT values, enabling the reconstruction of high‐resolution cross‐sectional images.^20^, ^21^ These reconstructed images are essential tools for clinicians during diagnosis and treatment planning. Static CT acquires complete projection data by combining multi‐source exposures with minor angle shifting of the gantry. All projection data in this study were acquired using a helical scanning mode.

### 2.2 Array‐type integrated x‐ray source

A key innovation of the static CT system lies in the integrated architecture of its x‐ray source, which seamlessly combines a high‐frequency inverter, x‐ray tube, and cooling unit into a single, self‐contained module. This high level of integration delivers substantial benefits, significantly improving the overall performance and practical utility of the system.

From a reliability standpoint, the all‐in‐one design eliminates the need for traditional high‐voltage cables and communication connectors typically found in separated component configurations. By removing these potential points of failure, the system achieves a notable boost in operational stability and fault tolerance. Moreover, the compact and lightweight structure of the integrated source allows for greater flexibility in installation, supporting high‐density deployment. This configuration not only ensures a uniform distribution of the array but also prevents stress concentration and promotes balanced load distribution across the system.

In terms of technical specifications, the integrated x‐ray source offers impressive output performance, providing a stable voltage of 120 kV and a current of 250 mA. The x‐ray tube employs thermionic emission. It delivers precise pulse control, with pulse widths adjustable down to 1 ms, and swift rise and fall times of under 10 microseconds, demonstrating exceptional capability for rapid switching. The reported values for these parameters are the mean values obtained from direct laboratory measurements. The system utilizes a sequential exposure mode, with a communication bus enabling precise delivery of commands to each module, allowing for fully independent operation of individual sources.

Each source supports an energy range of 70–140 kVp, which positions the system well for future development in multi‐energy spectral imaging. Additionally, the pulsed emission approach emulates the effect of high‐speed gantry rotation, greatly enhancing scanning efficiency. This not only improves image quality within shorter acquisition times but also enhances the scanning throughput of the machine. Compared with traditional spiral CT, the static CT system ultimately boosts the clinical workflow.

### 2.3 Photon stream detector

At the heart of the static CT system lies its photon stream detector, the primary component responsible for capturing x‐ray signals. This system utilizes photon stream detector modules selected for their high sensitivity and exceptional x‐ray conversion efficiency, allowing for the accurate detection of even low‐intensity photon emissions. Each module is designed with a finely tuned pixel pitch of 0.265 mm and features a resolution matrix of 288 × 160 pixels. A total of 64 such modules are arranged in a continuous circular configuration, forming the full detector ring. This geometric design ensures consistent angular coverage and leverages the superior properties of the detector materials to produce high‐fidelity imaging with enhanced detail.

A defining feature of this system is the fixed spatial relationship between the x‐ray sources and the detectors throughout data acquisition. Acquiring complete data requires only minor angle shifting. This minor angle shifting does not cause trailing and does not result in a decrease in spatial resolution. Furthermore, the reduced pixel size in the detector array plays a critical role in boosting spatial resolution, allowing for finer image detail.

### 2.4 Key correction and reconstruction algorithms

The static CT system features a distinctive dual‐ring configuration of x‐ray sources and detectors, a geometry that fundamentally departs from traditional cone‐beam CT systems. The source ring and detector ring of the static CT system have a longitudinal offset. Direct application of conventional cone‐beam reconstruction algorithms in this setup would likely result in image artifacts due to geometric incompatibility. To address this, we built upon the weighted extended Feldkamp–Davis‐Kress (xFDK) method introduced by Grimmer et al.,^22^ and developed an enhanced reconstruction algorithm, namely, the bilateral extended Feldkamp–Davis‐Kress (bixFDK) algorithm.

The bixFDK algorithm incorporates a refined weighting mechanism to account for the longitudinal displacement between the fixed‐position sources and detectors. This improvement enables more accurate volume reconstruction across the scan field, aligning the algorithm with the unique architecture of the static CT system. Building on this foundation, we further advanced the method into an iterative reconstruction framework, referred to as bixFDK‐IR. This compressed sensing‐based iterative approach, termed “iVision,” is particularly effective for sparse view scanning and can be applied to a broad range of anatomical regions.^23^

In addition to reconstruction, the absence of post‐collimators in the static geometry presents challenges for scatter suppression. To overcome this, we implemented a software‐based solution using the Weighted Fast Adaptive Scatter Kernel Superposition (w‐fASKS) technique.^24^ This method was further reinforced by integrating empirical scatter measurements derived from edge‐grating occlusion strategies.^25^ Together, these techniques offer robust correction of scatter artifacts across various anatomical areas, ensuring high image quality despite the system's non‐rotational design.

### 2.5 Control and workflow design

The operation of the static CT system is orchestrated through a centralized Modality Control System (MCS), which coordinates communication across multiple subsystems to carry out image acquisition and reconstruction tasks. Key components in this control framework include the Modality Reconstruction System (MRS), Patient Handling System (PHS), Reconstruction Task Manager (Recon), Control Box, and the Control Timing Box (CT Box), which manages high‐voltage activation and timing for the x‐ray sources.

**FIGURE 2:**
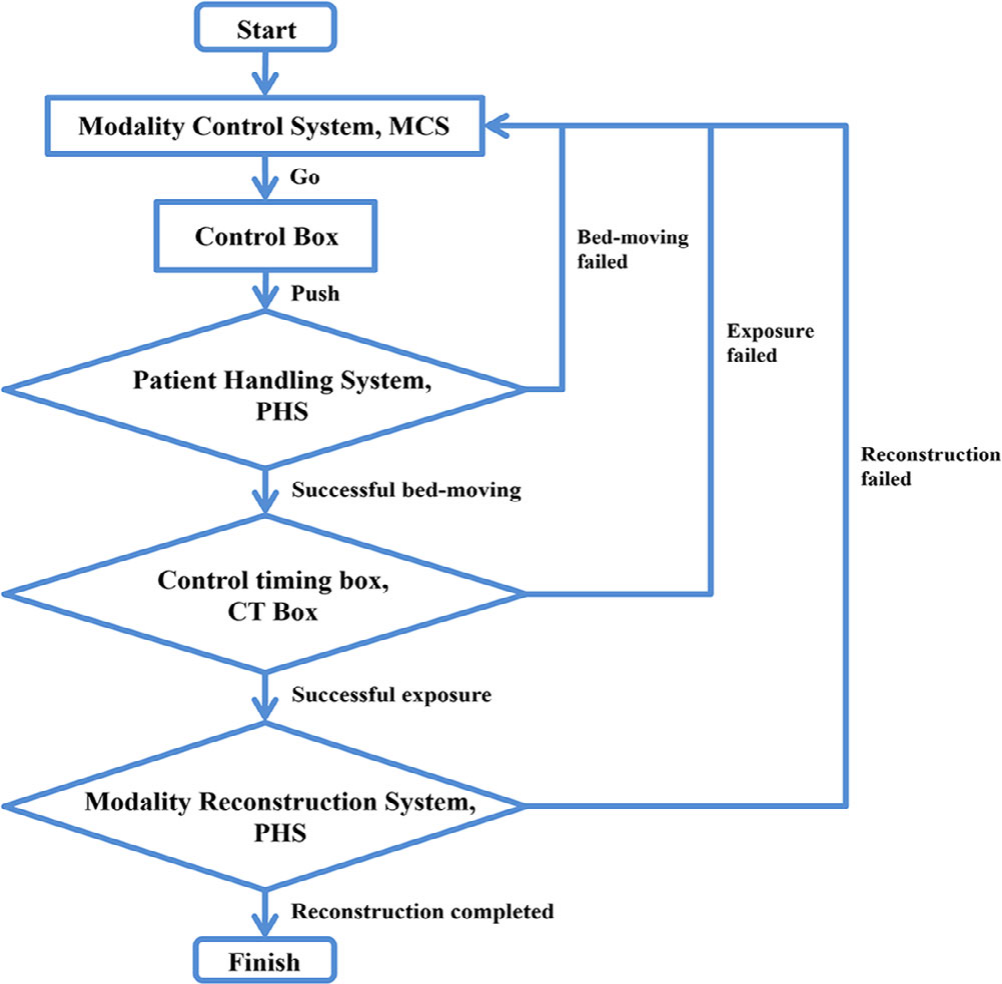
Simplified diagram of the control workflow.

As depicted in Figure 2, the full workflow is divided into three sequential stages: table positioning, image acquisition, and data reconstruction. The process begins when the technician selects the appropriate scanning protocol and inputs the relevant parameters through the MCS interface. Once these instructions are transmitted to the scanning table's Control Box, the technician initiates table movement by following prompts displayed on the interface.

After the PHS confirms the table is correctly aligned, the CT Box activates the high‐voltage supply and begins the x‐ray exposure phase. Upon completion of the scan, the MRS automatically triggers the reconstruction phase, managing the data flow to execute image processing and complete the full acquisition cycle.

The system includes built‐in safeguards to monitor each stage of the operation. Should any malfunction occur, whether during table movement, exposure, or reconstruction, the CT Box will immediately halt the process, preventing further execution and ensuring safe system operation.

## 3 RESULTS

### 3.1 Image validation of the static CT system

**FIGURE 3:**
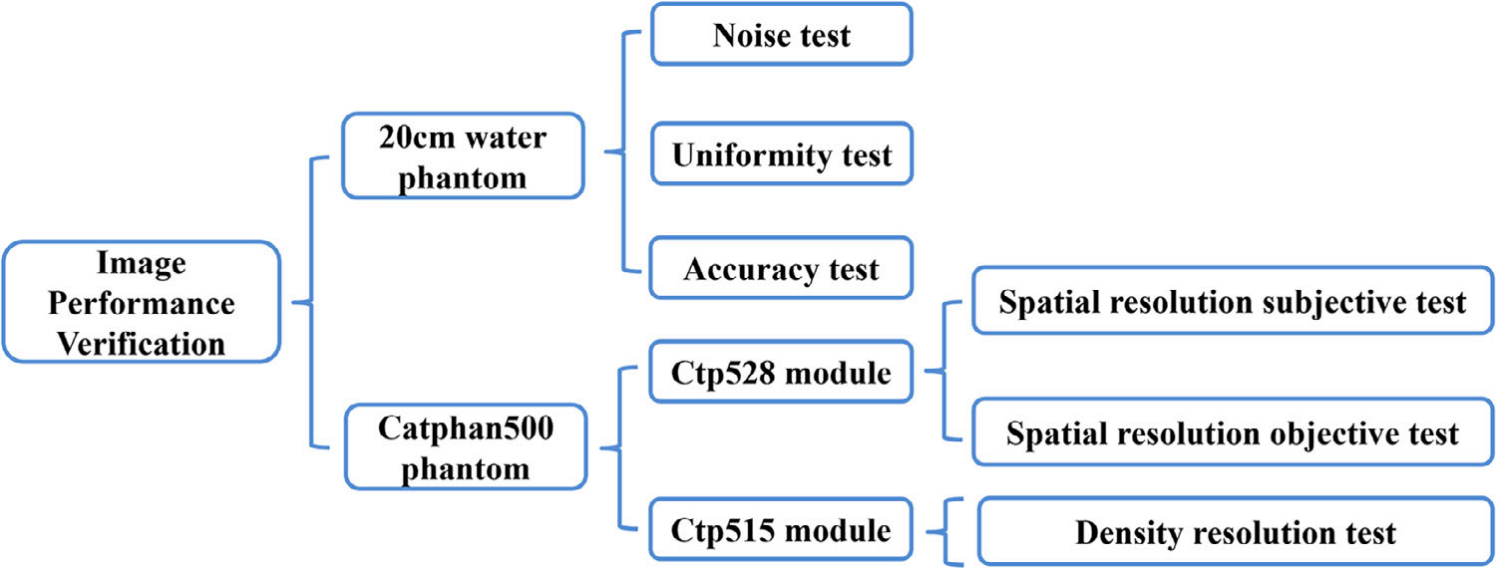
Experimental setup for image quality evaluation showing the phantoms and their corresponding test modules.

To assess the image reconstruction capabilities of the static CT system, five key image quality parameters were evaluated using a 20 cm water phantom along with the Ctp528 and Ctp515 modules embedded in the Catphan500 phantom (Figure 3). The water phantom, cylindrical with a 20 cm diameter, was employed to measure image noise, uniformity, and CT value accuracy. The Catphan500 phantom, constructed from solid acrylic, contains a spatial resolution module (Ctp528) composed of 21 sets of radially arranged high‐density line pairs, ranging from 1 LP/cm to 21 LP/cm, and a density resolution module (Ctp515), which includes three sets of contrast rods with attenuation differences of 1.0%, 0.5%, and 0.3%. Each set contains rods with diameters varying from 15.0 mm down to 2.0 mm. Scanning and reconstruction parameters are detailed in Table 1.

**TABLE 1:** Scanning and reconstruction parameters of phantom.

				Scanning parameters							Reconstruction parameters		
Scanned object		Tube voltage (kV)	Tube current (mA)	Acquisition time (s)	Puels time (ms)	View	Scanning mode	Focus mode	Pitch	FOV	Thickness (mm)	Reconstruction Kernel	CTDIvol (mGy)
Water phantom										250	10	Standard	19.2
Catpha‐n500	Ctp528	120	180	1.08	3	360	Helical scan	small focus	0.5	250	0.165	Sharp	19.1
	Ctp515									250	10	Smooth	18.5

Image quality testing was conducted in accordance with the IEC 61223‐3‐5 standard. Image noise was assessed by first reconstructing the CT scan, then placing a circular region of interest (ROI) covering roughly 40% of the water phantom's diameter. The standard deviation (SD) within this ROI was used to calculate noise via the equation: noise = (SD / 1000) × 100%.

To evaluate image uniformity, five smaller ROIs (each ∼10% of the phantom's diameter) were selected from different locations, and their mean CT values were compared. For accuracy, two ROIs, each with at least 100 pixels, were selected from the water and air regions, respectively, and their average CT values were computed.

Spatial resolution was evaluated both visually and quantitatively. In the visual method, the number of clearly distinguishable line pairs in the Ctp528 module was counted. Alternatively, Modulation Transfer Function (MTF) analysis was conducted to determine the spatial frequency at which the MTF dropped to 2%, offering an objective measure of resolution. A higher count of distinguishable line pairs or a greater MTF value indicated superior spatial resolution.

Density resolution was determined by identifying the smallest visible rod in the Ctp515 module. The smaller the visible rod, the better the system's ability to distinguish subtle density differences.

**FIGURE 4:**
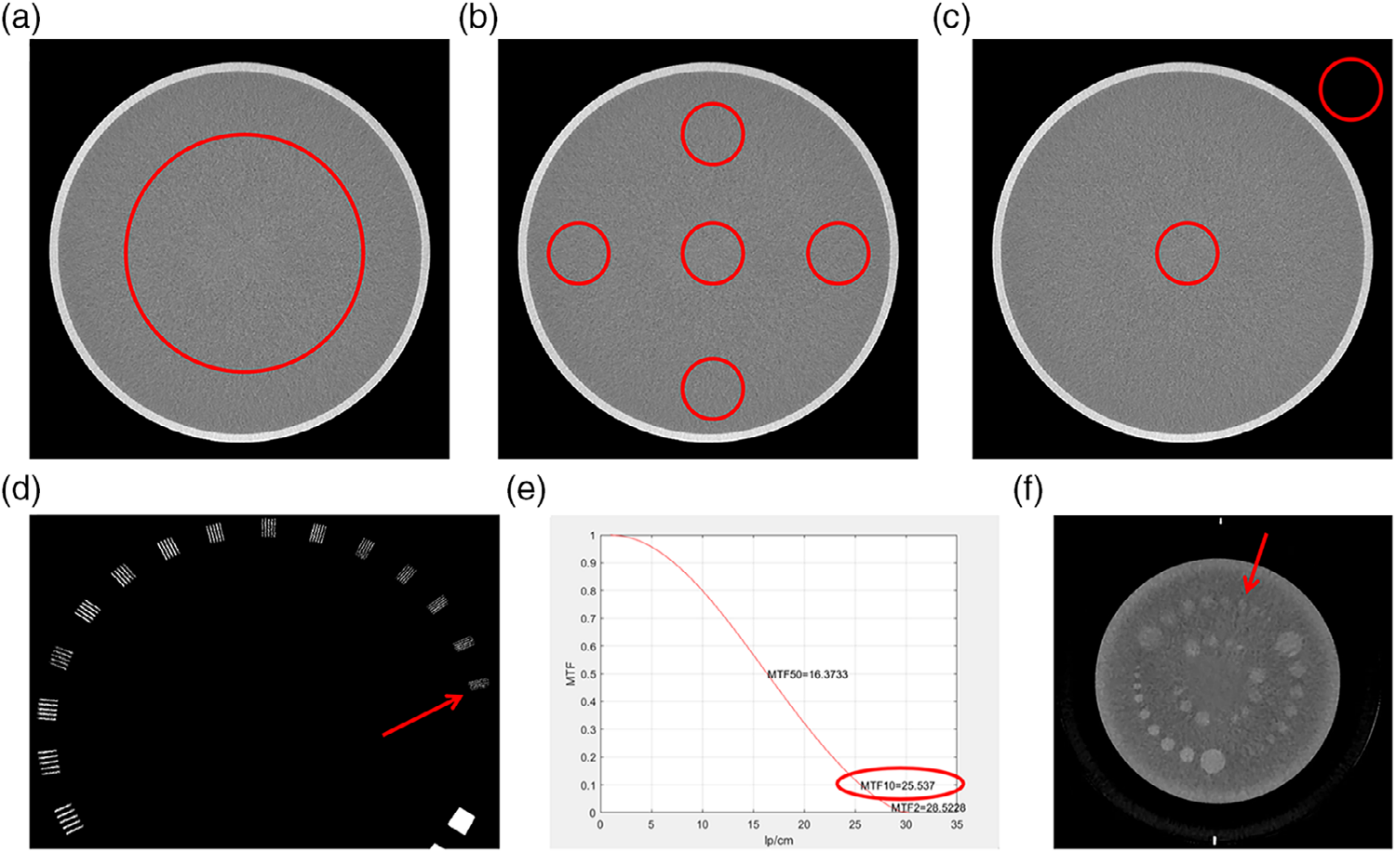
Water phantom with a diameter of 20 cm. (a) Noise; (b) Uniformity; (c) Accuracy results, standard Catphan500; (d) Subjective spatial resolution results; (e) Objective spatial resolution results; (f) Density resolution results.

Collectively, the results of these evaluations confirmed that the image quality of the static CT system meets the regulatory criteria outlined in IEC 61223‐3‐5 (see Figure 4 and Table 2).

**TABLE 2:** Image performance index.

Test item	Acceptance criteria	Test results
Noise (SD%)	≤0.35%	0.30
Uniformity (Hu)	≤±4	−2
Accuracy (Hu)	CT value (Water)≤0 ± 4	−2
	CT value (Air) ≤−1000 ± 10	−1005
Spatial resolution (Lp/cm)	Standard > 10.0 High resolution > 14.0	Subjective: 21 Objective: 25@MTF = 10%
Density resolution	–	3 mm @0.3%

### 3.2 Clinical experimental results

A volunteer study was conducted to evaluate the clinical performance of the static CT system. All participants provided written informed consent and the study received institutional review board approval.

**FIGURE 5:**
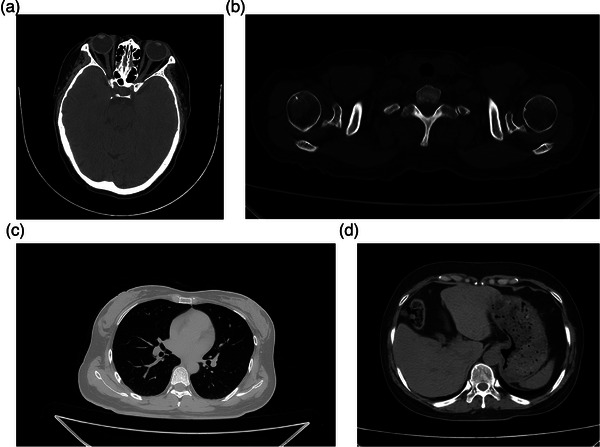
Clinical images. (a) Head; (b) Bones and joints; (c) Lung; (d) Abdomen.

Volunteers underwent CT scans targeting four anatomical regions: the head, bones and joints, chest, and abdomen (Figure 5). The head scans revealed fine anatomical structures, including the orbital ring and crystalline lens, with distinct visualization of the medial and lateral rectus muscles, as well as the ethmoid sinus (Figure 5a). In the bones and joints region, the imaging clearly captured the bone cortex, trabecular architecture, and cancellous bone of the humerus, without evidence of bone sclerosis artifacts (Figure 5b). Chest images provided detailed visualization of lung anatomy, including interlobar fissures, pulmonary textures, and both subpleural and extrapleural features (Figure 5c). Abdominal scans displayed clear organ boundaries, such as those of the liver, spleen, and stomach, with well‐defined surrounding fat structures (Figure 5d). Scanning and reconstruction parameters are detailed in Table 3. In static CT, the x‐ray tube operates with pulsed exposure, and the acquisition time refers to the duration of a single pulse exposure multiplied by the number of views per rotation.

**TABLE 3:** Scanning and reconstruction parameters of volunteer.

			Scanning parameters						Reconstruction parameters			
Scanned region	Tube voltage (kV)	Tube current (mA)	Acquisition time (s)	Pulse time (ms)	View	Scanning mode	Focus mode	Pitch	FOV	Thickness (mm)	Reconstruction Kernel	CTDIvol(mGy)
Head	120	180	1.35	2.5	540	Helical scan	Small focus	0.5	250	5	Smooth	52.53
Shoulder	120	200	0.864	3	288	Helical scan	Small focus	0.5	250	3	Smooth	16.98
Lung	120	200	0.72	2.5	288	Helical scan	Small focus	0.5	420	0.33	Sharp	14.15
Abdomen	120	200	0.72	2.5	288	Helical scan	Small focus	0.5	420	3	Smooth	14.15
Inner ear	120	180	0.9	2.5	360	Helical scan	Small focus	0.5	150	3	Smooth	35.02
Mediastinum	120	180	0.72	2.5	288	Helical scan	Small focus	0.5	420	3	Smooth	12.73
Hip joint	120	200	0.864	3	288	Helical scan	Small focus	0.4	420	3	Smooth	21.22
Ankle joint	120	90	0.72	2.5	288	Helical scan	Small focus	0.5	420	3	Smooth	6.37

**FIGURE 6:**
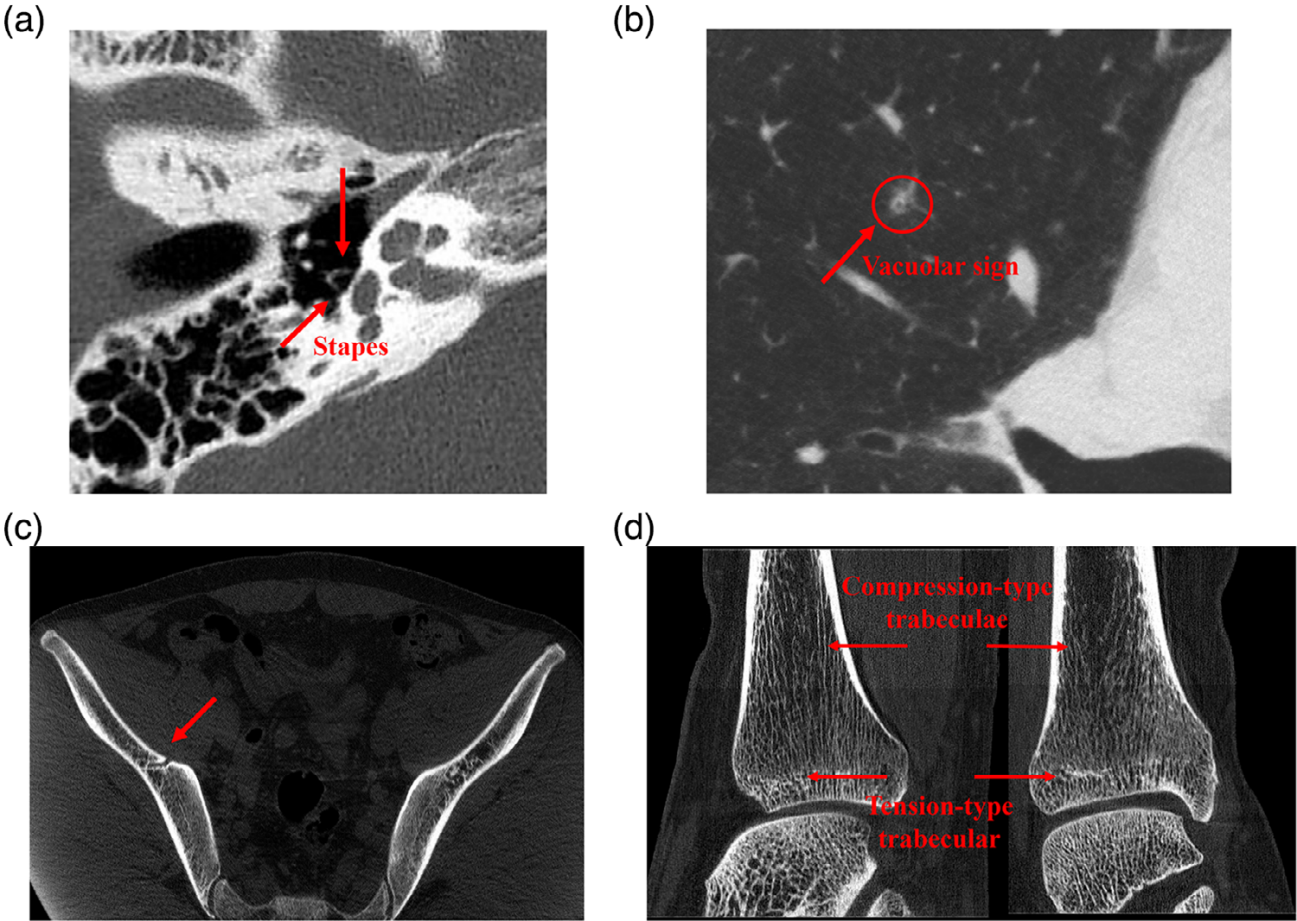
Clinical images. (a) Imaging of the stapes; (b) Pulmonary nodule; (c) Iliac bone microstructure; (d) Ankle joint trabecular bone.

Overall, the results demonstrated that the static CT system delivers high‐definition imaging across a range of clinically important regions. Moreover, subsequent clinical observations confirmed the system's superior performance in imaging bones and joints, and lung structures (Figure 6), reinforcing its potential for routine diagnostic applications.

## 4 DISCUSSION

The design of the static CT system is fundamentally driven by the need for multidimensional attenuation data in image reconstruction. By arranging multiple x‐ray sources uniformly along a circular path and enabling sequential short‐pulse emissions at fixed angles, the system achieves complete angular coverage only requires minor angle shifting. This scanning strategy not only eliminates motion‐induced blurring commonly seen in high‐speed rotational systems.

Beyond image quality, the system's dual‐ring geometry offers significant advantages from an engineering and manufacturing perspective. The symmetrical placement of internal components helps evenly distribute mechanical stress, minimizing vibration and ensuring operational stability. This mechanical balance contributes to the long‐term reliability of the system. Additionally, the use of standardized, modular components simplifies manufacturing workflows, enabling efficient use of uniform molds and reducing production costs. This streamlined approach enhances economic efficiency and boosts the system's market competitiveness.

The array‐type x‐ray source configuration enables precise and efficient acquisition of projection data from multiple angles, generating high‐resolution images suitable for clinical use. In practical applications, static CT has shown strong performance in diverse medical fields such as otolaryngology, respiratory diagnostics, and orthopedics. It enables the visualization of intricate structures like the stapes, cavitation features in pulmonary nodules, detailed profiles of hip fractures, and the complex architecture of trabecular bone, supporting both accurate diagnosis and advanced research.

In otolaryngology, the stapes,^26^, ^27^, ^28^, ^29^ a small, saddle‐shaped auditory ossicle, connects laterally with the incus and transmits sound vibrations from the middle ear to the cochlea via the oval window. Given its location behind the tympanic membrane and its small size, conventional CT often fails to depict it clearly without resorting to multi‐planar reconstruction (MPR).^30^, ^31^ Moreover, systems with inadequate spatial resolution may suffer from partial volume effects, further obscuring the stapes. By contrast, static CT delivers high‐resolution images that clearly display the stapes without requiring secondary scanning, demonstrating its strength in visualizing fine anatomical details.

In respiratory medicine, pulmonary nodules with cavitation signs, especially those under 5 mm in diameter, are critical indicators of malignancy.^32^, ^33^, ^34^, ^35^ Cavitation has been shown to correlate more strongly with malignancy than other morphological features such as lobulation or spiculation. Static CT effectively captures these subtle cavitation features, supporting early screening and diagnosis of malignant lung nodules.

In orthopedic applications, the increasing incidence of hip fractures, driven by population aging and frailty, has created a growing need for precise diagnostic imaging. Static CT provides surgeons with detailed visualization of fracture patterns, assisting in accurate preoperative planning.^33^ Furthermore, trabecular bone plays a central role in maintaining skeletal integrity.^36^, ^37^, ^38^, ^39^, ^40^ Its microstructure directly influences bone strength, stiffness, and overall biomechanical behavior. Static CT enables high‐resolution imaging of both tensile and compressive trabeculae and, when combined with post‐processing techniques, facilitates 3D reconstruction and quantitative analysis. This capability offers valuable insights into bone health and the pathophysiology of orthopedic diseases.

Static CT uses multiple sources and detectors. It needs only a small angle shifting to collect full projection data. The source ring and detector ring have a longitudinal offset. This offset often causes artifacts in traditional xFDK reconstruction. We improved xFDK by linking the offset to a height weight. This correction was added to the iterative weight function. The new method is called bixFDK. We tested the system using a 20 cm water phantom and a Catphan500 phantom. Image noise, uniformity, accuracy, and resolution were measured. Results confirm that all performance metrics meet requirements.

This study has several limitations. We did not scan the same volunteer with both static CT and traditional helical CT. Repeated scans would expose volunteers to extra radiation, which raised ethical concerns. The current evaluation relies on phantom experiments and preliminary comparisons with historical clinical data. Future studies will use specialized phantoms like Gammex and larger clinical trials. In CT image evaluation, multi‐planar reconstruction (MPR) of axial, coronal, and sagittal planes is a routine procedure. Images from different planes can display the spatial relationships of anatomical structures from a three‐dimensional perspective. However, this study relies solely on axial images for evaluation, which may lead to the omission of some anatomical details. In the future, we will conduct a more comprehensive study of static CT images.

## 5 CONCLUSION

In conclusion, static CT combines cutting‐edge imaging principles with practical clinical impact. Its superior image resolution, and broad applicability across medical disciplines position it as a critical tool in contemporary diagnostic imaging.

## CONFLICT OF INTEREST STATEMENT

The authors declare no conflicts of interest.

## ETHICS STATEMENT

The research protocol received approval from the Medical Ethics Committee of the First Affiliated Hospital of Soochow University (Approval ID: JD‐LX‐2021‐112), following the principles outlined in the 1964 Declaration of Helsinki and its later revisions. All participants provided written informed consent before undergoing imaging, and the entire study complied with institutional review board requirements.
